# Impacted Canines: Our Clinical Experience

**DOI:** 10.5005/jp-journals-10005-1111

**Published:** 2011-04-15

**Authors:** Sonia Chawla, Manoj Goyal, Karan Marya, Aakarsh Jhamb, Hind Pal Bhatia

**Affiliations:** 1 Reader, Department of Oral and Maxillofacial Surgery, Santosh Dental and Medical College Hospitals Ghaziabad, Uttar Pradesh, India; 2 Professor and Head, Department of Oral and Maxillofacial Surgery, Santosh Dental and Medical College Hospitals Ghaziabad, Uttar Pradesh, India; 3 Professor, Department of Oral and Maxillofacial Surgery, Santosh Dental and Medical College Hospitals Ghaziabad, Uttar Pradesh, India; 4 Reader, Department of Oral and Maxillofacial Surgery, Santosh Dental and Medical College Hospitals Ghaziabad, Uttar Pradesh, India; 5 Professor, Department of Pedodontic and Preventive Dentistry, Santosh Dental and Medical College Hospitals Ghaziabad, Uttar Pradesh, India

**Keywords:** Impacted maxillary canine, Impacted mandibular canine, Surgical exposure, Orthodontic repositioning.

## Abstract

**Background:**

To discuss the management of impacted canines and the various approaches used for the same.

**Materials and methods:**

The data of 33 cases, with 43 impacted canine teeth, seen and operated over a period of 3-year in Santosh Dental College and Hospital has been compiled. The diagnostic methods and treatment modalities undertaken are described and discussed.

**Results:**

Canine impactions were more common in the maxilla as compared with mandible in our study, which was statistically significant. Impacted canine position was mostly palatal in maxilla and labial in mandible. Chi-square test yielded a p-value of 0.002 which shows that there is an association between arch and position. The treatment options used were surgical exposure and orthodontic repositioning, cyst enucleation with extraction of impacted canine and surgical removal of impacted canine.

**Conclusion:**

Surgical exposure and orthodontic repositioning was successfully applied as first-line treatment for correcting ectopic positioned canine. In cases where exposure and subsequent orthodontic treatment was not indicated, the impacted canine was surgically removed to prevent future problems and surgical procedure was designed according to position of impacted canine.

## INTRODUCTION

Impacted canine is not an uncommon clinical problem in dental patients. The permanent canines are developed deep within the jaws, complete their development late, and emerge into oral cavity after the neighboring teeth. Due to these circumstances, eruption disturbances are more common with canines than with other teeth, except for third molar.^1^ Studies have reported that the incidence of tooth impaction varies from 5.6 to 18.8% of the population.^2-6^ It is of concern that the method of diagnosis and the choice of treatment planning of this problem poses as a dilemma to the dental clinician. Maximum success with least complications and failures requires a systematic approach to each case.

We have reviewed 34 patients diagnosed with impacted canines. Each of these case had a detailed history followed by meticulous clinical and radiological examination to determine the exact site and position of the impacted tooth and to detect if any associated pathology was present in relation to the impacted tooth. This paper describes our approach for management and highlights the tips for successful management which can be applied as a protocol to each patient presenting with impacted canine.

## MATERIALS AND METHODS

A total of 33 cases (13 males and 20 females) within the age group of 12 to 18 years, who were referred from the department of pedodontics, were included in this review. The criterion for a diagnosis of impacted canine included both clinical and radiological findings. The clinical elements which were suggestive of occurrence and location of impacted canine include:

1. Overretained deciduous canine.
2. Absent deciduous canine and resulting in loss of the available space for permanent tooth.
3. Tipping of lateral incisor in a vestibular or palatal position (Due to the pressure of the canine at its root).
4. Presence of a bump which can be felt by palpating the vestibular or palatal mucosa depending on the position of the retained canine. Clinically palpation of the buccal and palatal surface of the alveolar process distal to the lateral incisor may reveal the position of the maxillary canine about 1 to 1.5 years before emergence, and this has been suggested as a diagnostic tool.^1^

**Fig. 1:**
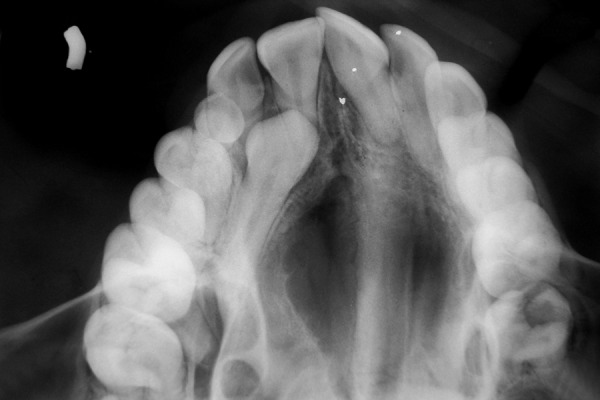
Occlusal view of maxilla showing impacted right maxillary canine

**Fig. 2:**
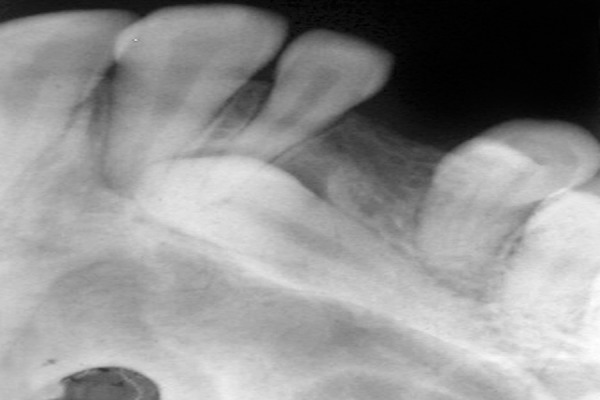
IOPA showing impacted maxillary canine

**Fig. 3:**
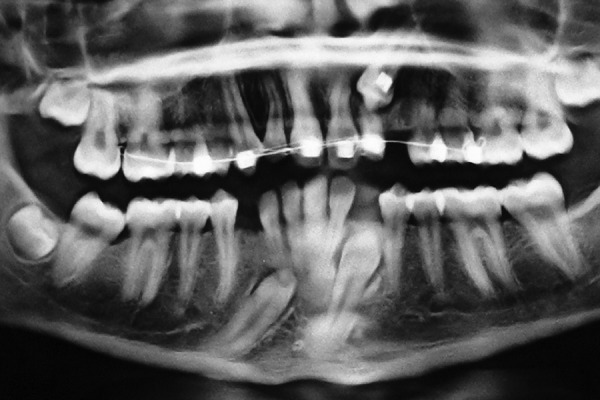
OPG showing bilateral impacted mandibular canines and left maxillary impacted canine

**Fig. 4:**
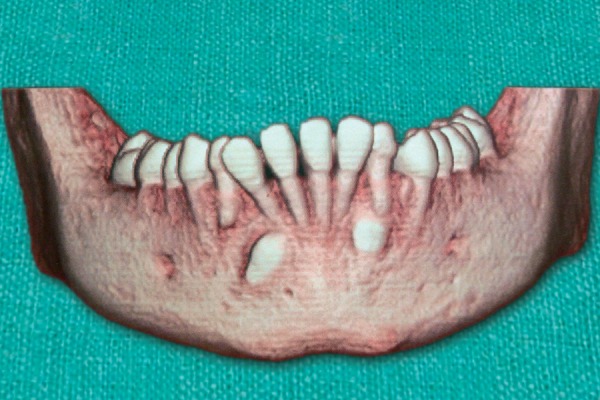
Dentascan showing bilateral impacted mandibular canines labially

This was followed by radiologic examinations (IOPA/ orthopantomogram/occlusal view/paranasal sinus view/ dentascan) which were indispensable in diagnosing and locating the position of impacted canines (Figs 1 to 4). The etiologic factors observed in our study were arch length, tooth size discrepancy, accounting for 41% of cases, followed by overretained deciduous teeth, early loss of deciduous canine, cyst, trauma, cleft palate and cleidocranial dysostosis.

The available treatment options were surgical removal of impacted canine (along with its associated pathology), or surgical exposure and orthodontic repositioning. Surgical removal of impacted canine was indicated if there was evidence of pathology around the tooth; if there was interference with planned orthodontic treatment; and if there was impingement on adjacent teeth. Exposure and orthodontic repositioning was carried out if space analysis revealed that tooth can be brought into occlusion and deviation of tooth axis was not too excessive (Table 1).

## RESULTS

**Graph 1:**
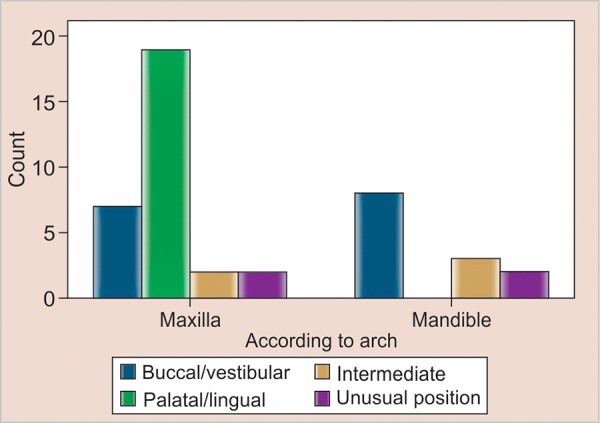
Arch *vs* position

Statistical tests were applied to the descriptive data to detect any predilection for either arch and any preponderance of placement within the arch. Z test for comparing proportions yielded p-value of 0.000123 which is significant. Impacted canines are most commonly found in maxilla as compared with mandible (Table 2). Chi-square test yielded a p-value of 0.002 which shows that there is an association between arch and position. In maxilla, the impacted canine is in palatal position whereas in mandible, it is commonly located in labial position (Tables 3, 4 and Graph 1).

**Fig. 5:**
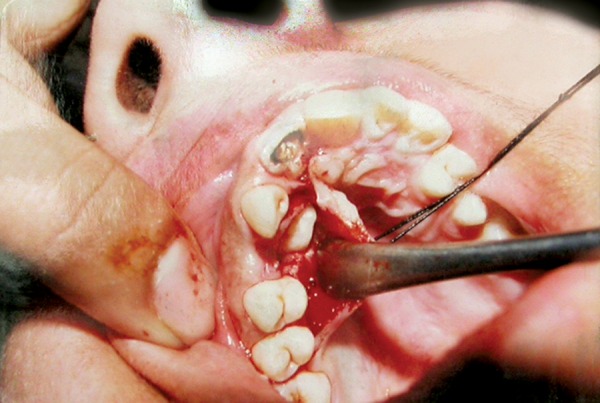
Clinical photograph showing palatal approach

**Fig. 6:**
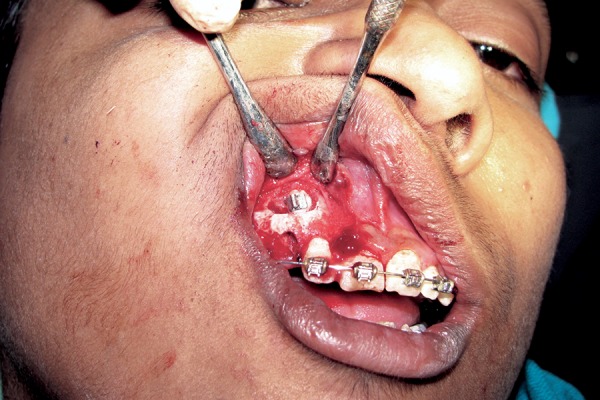
Clinical photograph showing buccal approach

The frequency of surgical approach was determined to show that the ease of access to the impacted canine is the main decisive factor (Figs 5 and 6). The operator should not be hesitant to use the combined approach wherever indicated (Tables 5 to 7).

## DISCUSSION

Canine impactions are more commonly associated with the maxilla than with the mandible; the same has been reported in our study which was statistically significant. One interesting finding seen in our study was the position of impacted canine in the two jaws that the impacted canine was mostly palatal in maxilla, whereas in the mandible it was more frequently seen in labial position. Chi-square test yielded a p-value of 0.002 which shows that there is a strong association between arch and position. According to Andreason,^1^ 85% impactions are palatally located in the maxilla. Unusual positions seen in our study was the location of canine in symphysis, maxillary sinus and infraorbital regions. Bilateral representation of impacted canines was also observed.

To establish a diagnosis, several diagnostic methods have been adopted which include a variety of radiographs. IOPA radiographs with or without shift cone technique was the primary diagnostic modality in all our cases. As per requirement other radiographs, such as orthopantomogram, occlusal and PNS views, were advised. Recently in few cases, computed tomography was utilized to evaluate the impacted canines. Dentascan provided detailed information about exact position of canine, degree of crowding, incisor resorption and width of dental follicle.^7-10^

**Fig. 7:**
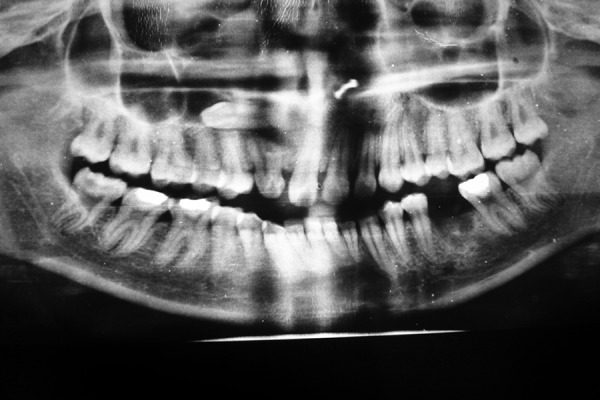
OPG showing horizontally impacted right maxillary canine

**Fig. 8:**
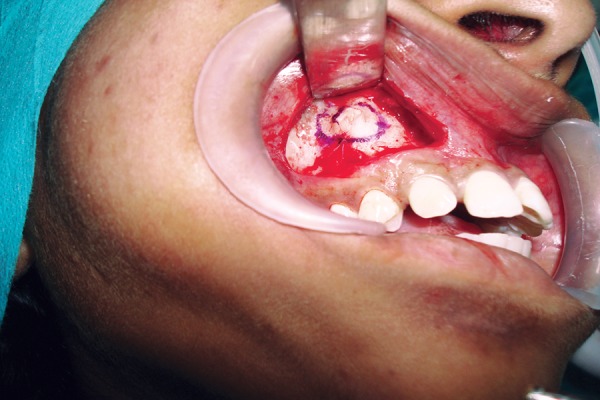
Flap raised and marking done

**Fig. 9:**
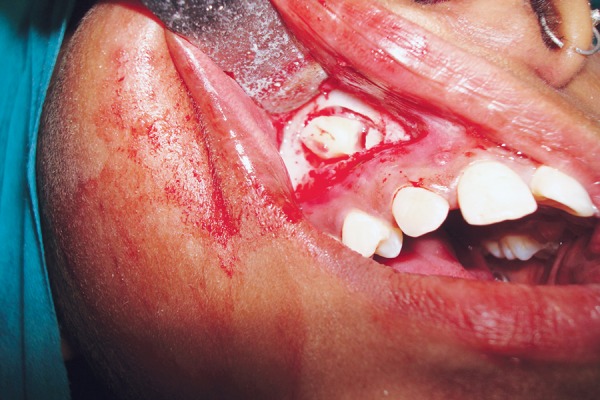
Impacted canine exposed and sectioned

**Fig. 10:**
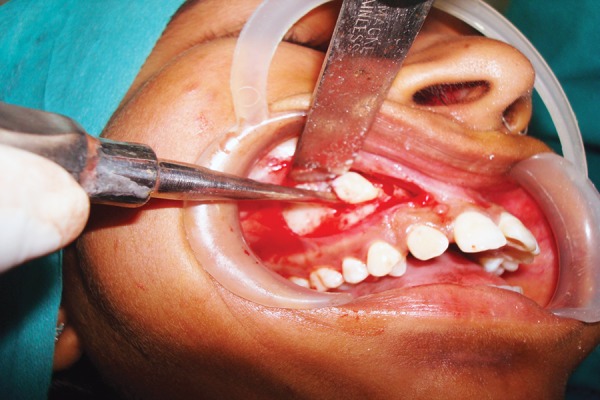
Removal of the tooth after sectioning

**Fig. 11:**
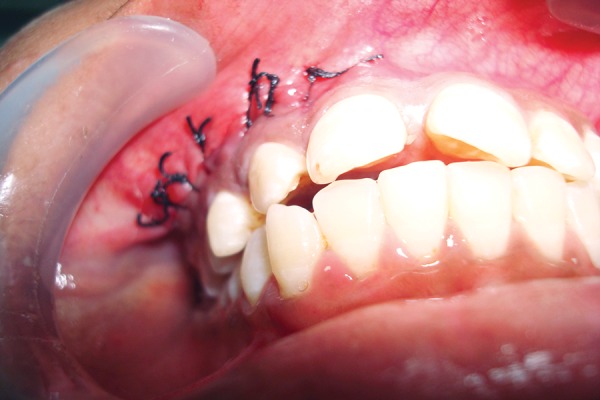
Primary wound closure

To treat a case of impacted canine, there can be various options ranging from keeping the patient under observation; surgical exposure and orthodontic repositioning; reimplantation and surgical relocation of impacted canine in the socket of the deciduous canine; to surgical removal of the impacted canine (Figs 7 to 11).

The factors that need to be evaluated before the mode of treatment of impacted tooth is decided, include the patient’s age, dental status of adjacent teeth (including periodontal, endodontic and operative status, shape and resorption), dental status of the impacted tooth, occlusal relationship, presence of any other associated condition (e.g. trauma/cyst/ odontoma) and arch length.^11-14^

Surgical exposure and orthodontic repositioning was considered the treatment of choice in all those cases wherever it was clinically feasible and a predictable and successful outcome could be obtained. The prognosis for orthodontically assisted eruption and repositioning of an impacted tooth within the alveolar process depends on the position and angulation of the impacted tooth, the length of treatment time, the patient’s age, degree of patient cooperation, the available space in the arch and the presence of keratinized gingival tissue.^15-17^

**Table 1:** **Table 1:** Treatment modalities

		*No. of cases* *(total 33)*		*Percentage*		*No. of teeth* *(total 43)*		*Percentage*	
Surgical removal of impacted canine		10		30.30		16		37.2	
Surgical exposure and orthodontic treatment		15		45.45		18		41.8	
Cyst enucleation with extraction of impacted canine		7		21.21		7		16.2	
Surgical removal with fracture fixation		1		3.03		2		4.6	

**Table 2:** **Table 2:** Position of impacted teeth

*Arch*		*No. of teeth (total 43)*		*Percentage*	
Maxilla		30		69.76	
Mandible		13		30.23	

**Table 3:** **Table 3:** Arch *vs* position crosstabulation

*Arch*				*Buccal/* * vestibular*		*Palatal/* * lingual*		*Intermediate*		*Unusual* * position*		*Total*	
Maxilla				7		19		2		2		30	
				23.3%		63.3%		6.7%		6.7%		100.0%	
Mandible		Count		8		0		3		2		13	
		Percent within		61.5%		0.0%		23.1%		15.4%		100.0%	
		according to arch											
Total				15		19		5		4		43	
				34.9%		44.2%		11.6%		9.3%		100.0%	

**Table 4:** **Table 4:** Statistical inference

		*Value*		*df*		*Asymp. sig.* *(two-sided)*	
Pearson Chi-square		14.870		3		0.002	
Likelihood ratio		19.700		3		0.000	
Linear-by-linear		0.020		1		0.887	
association							
No. of valid cases		43					

**Table 5:** **Table 5:** Surgical approaches

*Approach*		*No. of teeth*		*Percentage*	
Labial/vestibular		19		44.19	
Palatal		19		44.19	
Combined (labial and palatal)		5		11.63	
Total		43		100	

In cases, where exposure and subsequent orthodontic treatment was not indicated, the impacted canine was surgically removed to prevent future problems and surgical procedure was designed according to position of impacted canine.

After tooth impaction is diagnosed and if a decision to not undertake any immediate intervention is made, it is recommended that periodic observation is done to rule out possible pathological sequelae. During this observation period, clinicians must perform clinical and radiologic examinations after every 18 to 24 months. The following pathological sequelae associated with tooth impaction have been noted: Dentigerous cyst, odontogenic keratocyst, adenomatoid odontogenic tumor, calcifying epithelial odontogenic (Pindborg) tumor, odontogenic myxoma, ameloblastoma, external/internal resorption of the impacted tooth, external root resorption of adjacent teeth, transmigration, referred pain and periodontitis.^11,18-27^

Therefore, when decision is to retain an impacted tooth clinician must be vigilant for the potential development of a dentigerous cyst or ameloblastoma. The excised cyst/tissue should always be submitted to pathologist for microscopic examination for diagnosis.

**Table 6:** **Table 6:** Surgical approach *vs* position crosstabulation

						*Position*							
*Approach*				*Buccal/* *vestibular*		*Palatal/* *lingual*		*Intermediate*		*Unusual* *position*		*Total*	
Labial/				15		0		0		4		19	
vestibular				78.9%		0.0%		0.0%		21.1%		100.0%	
Palatal		Count		0		19		0		0		19	
		Percent within		0.0%		100.0%		0.0%		0.0%		100.0%	
		surgical approach											
Combined				0		0		5		0		5	
(labial				0.0%		0.0%		100.0%		0.0%		100.0%	
and palatal)													
Total				15		19		5		4		43	
				34.9%		44.2%		11.6%		9.3%		100.0%	

**Table 7:** **Table 7:** Statistical inference

		*Value*		*df*		*Asymp. sig.* *(two-sided)*	
Pearson Chi-square		86.000		6		0.000	
Likelihood ratio		83.591		6		0.000	
Linear-by-linear		7.749		1		0.005	
association							
No. of valid cases		43					

## CONCLUSION

In our study, canine impactions were more common in the maxilla as compared with the mandible which is statistically significant. Our study also shows that impacted canine position is mostly palatal in maxilla and labial in mandible. The crosstabulation shows that there is a strong association between arch and position. As the sample size was less, we need to study more number of cases to establish that such a correlation exists. Radiographs play a major role in diagnosing and determining the position of impacted canine. This further helps in deciding the approach to be used, i.e. labial, palatal or combined surgical approach. Various options can be used to manage a case of impacted canine. Surgical exposure and orthodontic repositioning was considered the treatment of choice. In cases where exposure and subsequent orthodontic treatment was not indicated, the impacted canine was surgically removed to prevent future problems and surgical procedure was designed according to position of impacted canine. We did not find any difference in complication rate between the palatal or buccal or combined approach.
